# Minding the gates: dual cell-autonomous and non-cell-autonomous mechanisms of TBK1 in ALS/FTD

**DOI:** 10.3389/fnins.2026.1941293

**Published:** 2026-09-24

**Authors:** Julie Smeyers, Daniel A. Mordes

**Affiliations:** Institute for Neurodegenerative Diseases, Weill Institute for Neurosciences, and Department of Pathology, University of California, San Francisco, San Francisco, CA, United States

**Keywords:** ALS, FTD, cell autonomous, non-cell-autonomous, *in vivo* models, TBK1

## Abstract

Amyotrophic lateral sclerosis (ALS) and frontotemporal dementia (FTD) comprise a neurodegenerative disease spectrum characterized by TDP-43 pathology and overlapping genetic risk factors. Loss-of-function variants in *TBK1* (TANK-binding kinase 1) represent one of the most common genetic causes of ALS/FTD, highlighting haploinsufficiency for TBK1 as a major contributor to disease pathogenesis. Emerging evidence from iPSC and mouse models suggests that reduced TBK1 activity disrupts interconnected autophagy, endo-lysosomal, and innate immune pathways through both cell-autonomous and non-cell-autonomous mechanisms. In this review, we discuss the multifaceted role of TBK1 as a master regulator of proteostasis and inflammatory pathways, highlight its critical role in maintaining microglial homeostasis, and explore how these convergent functions inform therapeutic strategies for ALS/FTD.

## Introduction

### TBK1 variants in the ALS/FTD disease spectrum

Amyotrophic lateral sclerosis (ALS) and frontotemporal dementia (FTD) occur on a shared neurodegenerative disease spectrum with overlapping clinical, pathological, and genetic features. FTD is characterized by the degeneration of cortical neurons within the frontal and temporal lobes, leading to gradual behavioral, cognitive, and language impairments (Neary et al., 2005). In ALS, rapidly progressive degeneration primarily affects upper and lower motor neurons within the motor cortex, brainstem, and ventral spinal cord, resulting in severe muscle atrophy and respiratory failure (Feldman et al., 2022). Almost all (~97%) of ALS and about half of FTD patient brains harbor pathological inclusions of the RNA-binding protein TDP-43 (Neumann et al., 2006; Mackenzie and Rademakers, 2008). Despite tremendous progress, disease-modifying treatments are not available for ALS/FTD associated with TDP-43 pathology. While the vast majority of cases are sporadic, a family history is present in only about 10% of ALS patients, with a complex genetic landscape of more than 30 causal or risk-associated genes identified to date (Al-Chalabi et al., 2024). While variants in some genes are specifically linked to one disease, such as *SOD1* or *FUS* in ALS, and *MAPT* or *GRN* in FTD, many genes associated with ALS/FTD (including *C9orf72*, *TBK1*, *SQSTM1*, *OPTN*, and *VCP*) are implicated in the regulation of autophagy and innate immunity, suggesting that disruptions in these cellular pathways contribute to disease pathogenesis (Ranganathan et al., 2020).

Whole-exome sequencing (WES) studies in both familial and sporadic patient cohorts have consistently linked TBK1 to neurodegeneration (Cirulli et al., 2015; Freischmidt et al., 2015; Gijselinck et al., 2015; Le Ber et al., 2015; Pottier et al., 2015; Williams et al., 2015; Borghero et al., 2016; Shu et al., 2016; Tsai et al., 2016; van der Zee et al., 2017; Foster et al., 2020), in which loss-of-function (LOF) *TBK1* variants represent the third most common genetic cause of combined familial ALS/FTD in certain cohorts. Many disease-associated variants primarily act in a heterozygous dominant manner, as nonsense or frameshift mutations, leading to reduced levels of functional TBK1 protein, consistent with *TBK1* haploinsufficiency. Neuropathological studies of TBK1 variant carriers have predominantly reported TDP-43 type B proteinopathy, with TDP-43 cytoplasmic inclusions in cortical and in lower motor neurons of the spinal cord, yet other TDP-43 pathological types as well as p62-positive cytoplasmic inclusions have also been described (Freischmidt et al., 2015; Pottier et al., 2015; van der Zee et al., 2017; Domoto-Reilly et al., 2024). Interestingly, accumulation of phosphorylated TBK1 has been observed in the frontal cortex of C9ORF72-FTD patients, raising the possibility that impaired TBK1 function may contribute to TDP-43 pathology beyond TBK1-associated ALS/FTD (shao et al., 2022). Deleterious missense variants occur across protein-coding regions, suggesting that the neurodegenerative phenotype is driven not only by a loss of kinase catalytic activity but also by the disruption of critical protein–protein interactions, though some variants remain of unknown clinical significance (de Majo et al., 2018; Ye et al., 2019). For example, pathogenic coding variants within a key C-terminal region, such as E696K or L683P, affect the interaction between TBK1 and the autophagic cargo receptor optineurin (OPTN), which is also linked to familial ALS and ALS/FTD (Pottier et al., 2015; Freischmidt et al., 2017).

### Human genetics link TBK1 to additional diseases

Genetic studies have linked TBK1 to a wider spectrum of distinct clinical phenotypes. For instance, homozygous LOF variants can lead to a severe systemic autoinflammatory syndrome, independently of vulnerability to viral pathogens (Taft et al., 2021). Heterozygous LOF variants impair TLR3-mediated immunity and confer susceptibility to childhood herpes simplex encephalitis (Herman et al., 2012). TBK1 has been implicated in innate immune signaling as a key regulator of interferon (IFN-I) production and downstream inflammatory responses that are implicated in autoimmune disorders (Sugrue et al., 2021). Additionally, multiple sclerosis (MS) patients exhibit marked upregulation of *TBK1* in immune cell populations, and an MS susceptibility locus maps to *TBKBP1*, which encodes a TBK1-interacting protein (Sorosina et al., 2022). On the other hand, copy number variations leading to *TBK1* gene duplication are associated with normal-tension, open-angle glaucoma (Minegishi et al., 2013; Ritch et al., 2014), which may be associated with dysregulation of autophagy in retinal ganglion cells (McElnea et al., 2014). While somatic variants have not been associated with cancer, overexpression and aberrant activation of TBK1 are implicated in the oncogenesis of several malignancies, including forms of leukemia (Runde et al., 2022).

### TBK1 protein structure and activation mechanisms

TBK1 (TANK-binding kinase 1), also designated as NAK or T2K, is a ubiquitously expressed serine/threonine kinase belonging to the IKK family of protein kinases (Pomerantz and Baltimore, 1999; Peters et al., 2000; Fitzgerald et al., 2003). TBK1 acts as a master regulator of diverse cellular processes, including proliferation, apoptosis, metabolism, innate immunity, and autophagy (Xu et al., 2018; Miranda et al., 2024; Wisessaowapak et al., 2026) (Figure 1). Structurally, the monomeric protein is organized into three major functional regions: an N-terminal serine/threonine kinase domain (KD) containing a regulatory activation loop, a central ubiquitin-like domain (ULD), and a C-terminal scaffolding region comprised of two coiled-coil domains (CCD1 and CCD2). TBK1 functions as a homodimer, though it can also form heterodimers with the closely related kinase IKKε (Tu et al., 2013). Activation of the TBK1 dimer requires clustering, facilitated by the local accumulation of polyubiquitin chains that promote trans-autophosphorylation at serine 172 (S172) within the kinase domain activation loop. Distal to the alpha-helical scaffold and dimerization domain (SDD), the critical CCD2 region recruits specific regulatory adaptors, including TANK, AZI2/NAP1, and TBKBP1/SINTBAD (Ma et al., 2012; Helgason et al., 2013; Larabi et al., 2013). These adaptor complexes do not simply modulate kinase activity; they dictate the spatial configuration of the complex, guiding TBK1 to distinct subcellular compartments to selectively engage either autophagic or innate immune signaling cascades (Endo et al., 2024).

**Figure 1:**
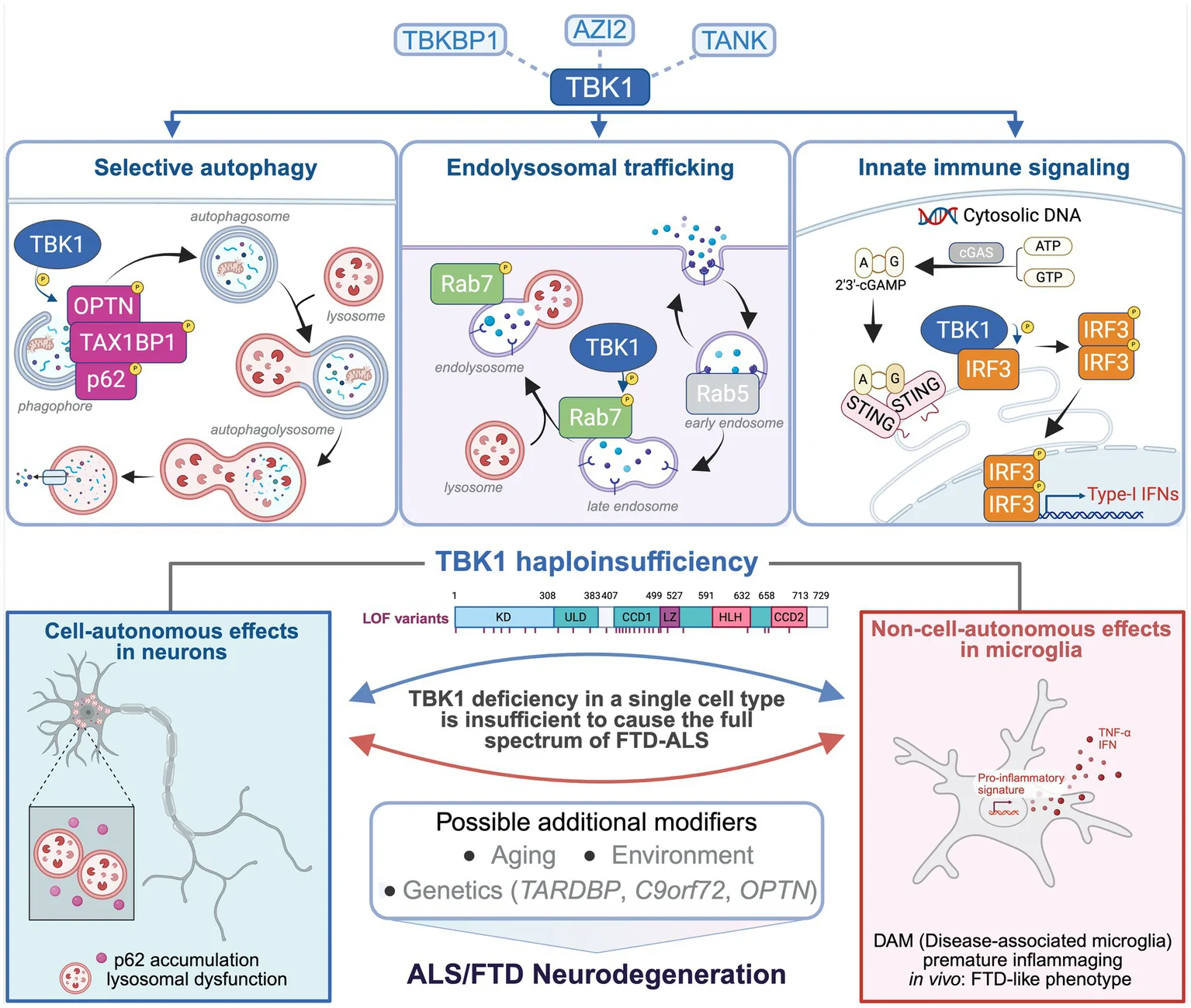
TBK1 integrates neuronal proteostasis and glial immune homeostasis in ALS/FTD. TBK1 functions as a central signaling hub integrating selective autophagy, endo-lysosomal trafficking, and innate immune pathways. Through interactions with adaptor proteins including TBKBP1, AZI2/ NAP1, and TANK, TBK1 is recruited to distinct cellular compartments to regulate cargo receptor-mediated selective autophagy, lysosomal homeostasis, and type I interferon signaling. ALS/ FTD-associated TBK1 loss-of-function variants (KD: Kinase Domain, ULD: Ubiquitin-like Domain, CCD1: Coiled-coil Domain 1, LZ: Leucine Zipper, HLH: Helix–Loop–Helix, CCD2: Coiled-coil Domain 2) result in haploinsufficiency and disrupt these interconnected pathways. In neurons, reduced TBK1 activity impairs proteostasis, autophagic clearance, and endosomal trafficking, producing cellular vulnerabilities. In contrast, TBK1 deficiency in microglia alters immune homeostasis, promoting pro-inflammatory states that contribute to neurodegeneration through non-cell-autonomous mechanisms. Additional risk factors and stressors, including aging, genetic variants, or environmental insults, may be required to drive progression towards ALS/FTD characterized by TDP-43 pathology and gradual loss of susceptible excitatory neurons.

## TBK1 at the intersection of key cellular pathways

### TBK1 orchestrates selective autophagy

While macroautophagy non-selectively engulfs cytoplasmic content for nutrient recycling, selective autophagy targets specific ubiquitinated substrates, including misfolded proteins and damaged organelles, for lysosomal degradation (Zaffagnini and Martens, 2016; Gatica et al., 2018; Griffey and Yamamoto, 2022). TBK1 promotes this concerted process through the regulation of selective autophagy factors at several steps (Herhaus et al., 2019; Harding et al., 2021; Manganelli et al., 2026; Mordes and Smeyers, 2026). First and foremost, TBK1 directly phosphorylates autophagy cargo receptors, including p62/*SQSTM1*, OPTN, NDP52/CALCOCO2, and TAX1BP1, markedly increasing their binding affinity for ubiquitinated substrates, including damaged mitochondria (Richter et al., 2016; Oakes et al., 2017; Adriaenssens et al., 2024). For instance, TBK1-mediated phosphorylation of OPTN enhances its binding to both polyubiquitinated substrates and ATG8/LC3 proteins, directly anchoring the targeted cargo to the expanding autophagosomal membrane. During autophagy pathway initiation, TBK1 has been implicated in driving autophagosome biogenesis by regulating syntaxin17 and early assembly complexes (Jung et al., 2009; Kumar et al., 2019; Vargas et al., 2019) while simultaneously modulating autophagy flux by phosphorylating GABARAPL2 to dismantle the initiation hub and prevent excessive pathway activation (Gestal-Mato et al., 2026).

### TBK1 as a central regulator of innate immune signaling

TBK1 is a critical signaling hub in innate immune signaling, acting as an integrator of multiple upstream pattern recognition receptor (PRR) pathways: 1) the cGAS-STING axis activated by cytosolic double-stranded DNA (dsDNA); 2) the RIG-I-like receptor (RLR/MAVS) pathway triggered by double-stranded RNA, and 3) the TRIF-dependent Toll-like receptor signaling responding to endosomal dsRNA (TLR3) or extracellular LPS (TLR4). All of these pathways converge on TBK1 activation (Kagan et al., 2008; Liu et al., 2015). Once activated, TBK1 phosphorylates STING, IRF3, and IRF7, prompting their nuclear translocation and inducing interferon (IFN-I) synthesis (Tanaka and Chen, 2012; Liu et al., 2015; Zhao et al., 2019). TBK1 also cooperates with IKKε to promote the activation of the pro-inflammatory transcription factor NF-κB (Fitzgerald et al., 2003; TenOever et al., 2007; Fitzgerald and Kagan, 2020). Finally, TBK1 acts as a molecular checkpoint in the NLRP3 inflammasome pathway, serving as an “off-switch” to limit hyperactivation, preventing premature inflammasome assembly, chronic cytokine release, and pyroptotic cell death (Fischer et al., 2021).

### TBK1 regulates endosomal trafficking and lysosomal homeostasis

Beyond its established roles in selective autophagy and innate immunity, TBK1 serves as an important regulator of the endo-lysosomal network, orchestrating vesicle trafficking, early-to-late endosomal maturation, and lysosomal quality control. In neurons, TBK1 is implicated in RAB5-mediated early endocytic vesicle transport and maturation (Lu et al., 2020; Shao et al., 2022). Subsequently, TBK1 modulates late endosomal dynamics by phosphorylating the late endosomal GTPase RAB7 at serine 72 (S72), a key post-translational modification that dictates RAB7 binding to cytoplasmic dynein motor adaptors (Heo et al., 2018; Smeyers et al., 2025; Villarroel-Campos et al., 2026). Interfering with this regulatory axis compromises the normal retrograde transport of signaling endosomes, leading to severe bidirectional transport defects that selectively disrupt the trafficking of signaling endosomes while sparing the axonal transit of mature lysosomes and mitochondria (Villarroel-Campos et al., 2026). Downstream of vesicle transport, TBK1 activity directly sustains lysosomal membrane integrity and luminal proteolytic capacity. Complete loss of TBK1 protein expression results in increased accumulation of acidic vesicles, yet a marked decrease in lysosomal protease cathepsin B activity, suggesting significant impairment of endo-lysosomal function (Smeyers et al., 2025). When lysosomal membranes undergo physical or chemical damage, ubiquitin-mediated signaling drives the localized recruitment of TBK1 and selective autophagy cargo receptors, including OPTN and TAX1BP1, directly to the injured membranes to clear damaged lysosomes, termed lysophagy (Eapen et al., 2021). Consequently, genetic ablation or small-molecule inhibition of TBK1 completely blocks lysophagic flux in both HeLa cells and human iPSC-derived neurons, establishing another role for TBK1 as a key gatekeeper of lysosomal quality control (Eapen et al., 2021).

### Crosstalk: the interconnection between selective autophagy and innate immunity

TBK1 is broadly expressed across the central nervous system (CNS) and peripheral immune cell types, including macrophages and microglia (Li et al., 2024a). In myeloid cells, TBK1 sits at the center of crosstalk between innate immunity and endo-lysosomal pathways, coordinating the balance between homeostatic and pro-inflammatory states. Immune activation directly prompts TBK1 and its upstream regulators, such as TRIM23, to initiate selective autophagy (Sparrer et al., 2017; Nozawa et al., 2020). This cascade drives both xenophagy against pathogens and the TAX1BP1-mediated clearance of inflammatory MAVS aggregates to dampen prolonged antiviral signaling (White et al., 2025). Additionally, the autophagy machinery provides feedback control to prevent hyperinflammation. The cargo receptor p62/SQSTM1 directly inhibits the TBK1-IRF7 pathway to suppress excessive type I interferon production (Prabakaran et al., 2018; Li et al., 2024b), while the ubiquitin ligase NEDD4 targets TBK1 for NDP52-mediated autophagic degradation during late-stage infection to resolve immune response and restore cellular homeostasis (Xie et al., 2022). Furthermore, the dual-role cargo receptors OPTN and TNIP1, both substrates of TBK1 and independently linked to ALS/FTD pathogenesis, physically bridge these pathways. Beyond driving cargo-specific selective autophagy, OPTN and TNIP1 actively inhibit cGAS-STING signaling to suppress chronic inflammation (Padman et al., 2019; Le Guerroué et al., 2023).

## The glial perspective of TBK1 in ALS/FTD: insights from *in vivo* and iPSC models

### Neuroinflammation and glial activation in ALS/FTD

Neuroinflammation in ALS/FTD is characterized by widespread glial activation and an innate immune response that can be measured in patients. *In vivo* Positron Emission Tomography (PET) imaging targeting TSPO (translocator protein 18 kDa), a marker of glial activation, demonstrates extensive neuroinflammation across the frontal, prefrontal, motor, and temporal cortices (Zhang, 2015; Bevan-Jones et al., 2019; Malpetti et al., 2021). Pathological analysis corroborates this regional burden, showing profound astrogliosis and microgliosis (CD68 + and IBA1 + reactive profiles) restricted to areas of active motor neuron loss, while completely sparing non-degenerating regions, such as the dorsal (sensory) horns of the spinal cord (Philips and Robberecht, 2011; Brettschneider et al., 2013; Rifai et al., 2022). Similarly, fluid-based biomarker studies demonstrate elevated cerebrospinal fluid (CSF) and plasma levels of pro-inflammatory cytokines (IL-6, TNF-α), macrophage-derived chemokines (MCP-1), and chitinases (CHIT1, YKL-40) in patients that track with disease progression (Kuhle et al., 2009; Gaur et al., 2020; Masrori et al., 2022). Consistent with functional involvement of glia, gene expression analysis reveals that many ALS/FTD risk genes, including *TBK1, C9orf72*, and *GRN*, are differentially expressed across CNS cell types, with substantial expression in microglia and/or astrocytes (Hashimoto et al., 2022). Together, these imaging, histological, and fluid metrics provide quantifiable evidence of a coordinated, widespread neuroinflammatory response concomitant with neurodegenerative processes.

### Cell-autonomous role of TBK1 in ALS/FTD

Systemic homozygous *Tbk1* knockout causes embryonic lethality in mice due to massive TNF- α -dependent liver necrosis (Bonnard et al., 2000; Xu et al., 2018). Consequently, heterozygous *Tbk1* (*Tbk1*^+/−^) mice, which may reflect haploinsufficiency found in ALS/FTD variant carriers, bypass this lethality. However, this partial depletion of Tbk1 is insufficient to either alter cognitive performance or induce motor neuron degeneration in aging mice (Bruno et al., 2020). Similarly, while complete absence of TBK1 in human iPSC-derived neurons disrupts protein homeostasis and RAB7 phosphorylation, it does not appear to precipitate TDP-43 mislocalization or downstream disease-associated cryptic splicing events, as shown by the absence of *STMN2* cryptic exon induction (Smeyers et al., 2025; Mordes and Smeyers, 2026). Several studies have closely examined cellular and murine models harboring disease-associated variants (Supplementary Table S1). For instance, the *TBK1 E696K* mutation, which disrupts the TBK1-OPTN complex while retaining TBK1 kinase activity, results in abnormal, nutrient-insensitive retention of the kinase on lysosomal membranes (Brenner et al., 2024; Talaia et al., 2024), thereby driving increased RAB7 phosphorylation and aberrant mTORC1 signaling. *In vivo*, this point mutation leads to the accumulation of p62 and the Atg8-related autophagy protein GABARAPL1 in cytoplasmic inclusions and is associated with progressive, age-dependent motor deficits, despite the absence of detectable TDP-43 pathology. Thus, disruption of this key TBK1-OPTN interaction can drive motor dysfunction despite preserved TBK1 kinase activity, although this model does not recapitulate the full pathological spectrum of ALS/FTD (Brenner et al., 2024). In contrast, animals harboring variants associated with partial loss of kinase activity, such as the *Tbk1 G217R* mutant allele, or with motor-neuron-specific conditional knockout of *Tbk1* do not exhibit neurodegeneration (Gerbino et al., 2020) (Supplementary Table S1). Similarly, mice carrying the *R228H* mutation, which impairs TBK1 kinase function, are phenotypically normal. However, when exposed to a proteotoxic stressor, defects in endosomal homeostasis associated with protein aggregation are exacerbated, highlighting that reduced TBK1 activity can compromise neural proteostasis (Shao et al., 2022). Unmasking further phenotypic effects downstream of TBK1 loss of kinase activity ultimately requires an additional trigger, such as overexpression of mutant SOD1-G93A, to explicitly cause neuron loss *in vivo* (Gerbino et al., 2020) (Figure 1).

### Non-cell-autonomous contribution of glia to ALS/FTD

The non-cell-autonomous contributions of glia to the pathogenesis of ALS/FTD are attracting increasing attention. While neurons remain primarily vulnerable to cell-autonomous endo-lysosomal trafficking defects, myeloid-specific *Tbk1* deletion gives rise to an FTD-like behavioral phenotype, characterized by impaired social recognition, elevated microglial density, and localized recruitment of cytotoxic (CD8^+^) T lymphocytes to the substantia nigra pars reticulata (Lenoel et al., 2025) (Supplementary Table S1). Under homeostatic conditions, loss of TBK1 does not induce major transcriptomic alterations in microglia. However, following acute immune stimulation with lipopolysaccharide (LPS) or poly I:C, primary murine microglia exhibit a dampened pro-inflammatory and type I interferon response while simultaneously upregulating disease-associated microglia (DAM) markers, including *Itgax*, which is also elevated in ALS patients (Lenoel et al., 2025; Zhang et al., 2026). This DAM-like phenotype is observed in isolated microglia, where transcriptomic analyses reveal increased expression of *Itgax*, *Cst7*, and *Axl*, suggesting compensatory responses to defects in phagocytosis or lysosomal pathways due to TBK1 deficiency. Likewise, a constitutive heterozygous *Tbk1* KO drives a chronic, inflammaging-like baseline immune signature (Bruno et al., 2020), mimicking an aged immune state that might prime the CNS for neurodegenerative processes. This is mirrored in patients, where isolated peripheral blood mononuclear cells (PBMCs) and monocytes from ALS cohorts similarly exhibit a marked reduction in phosphorylated TBK1 (Duan et al., 2021), underscoring that a functional myeloid signaling deficit may actively accompany the disease state. To cross the threshold for initiation of a neuropathological cascade, additional genetic, environmental, and/or age-related catalysts are likely required. One model proposes that aging contributes to the systemic decline of the kinase TAK1, a regulator of immune pathways that serves as an inhibitor of another kinase, RIPK1, in myeloid cells. When TAK1 is deleted in the background of *Tbk1* deficiency, the resulting hyperactivation of RIPK1 drives several ALS/FTD-related pathological features, including neuroinflammation, TDP-43 aggregation, and axonal loss (Xu et al., 2018). On the other hand, acute physical trauma may potentiate the effects of TBK1 haploinsufficiency since post-injury TBK1 inhibition shifts microglia into a highly reactive, DAM profile (Rehman et al., 2021). Intersecting these models with the classic *SOD1^G93A^* background further highlights this temporal duality, where partial TBK1 protein loss in all cell types accelerates early disease onset likely by impairing the autophagy pathway in motor neurons, but paradoxically prolongs survival in late-stage disease by dampening terminal hyperinflammation (Brenner et al., 2019). Collectively, these discoveries converge onto multi-component mechanisms closely mimicking those of the *C9orf72* gene. Much like the multifaceted roles of TBK1, the widely studied ALS/FTD-associated protein C9orf72 principally regulates endosomal trafficking and the autophagy pathway in neurons, yet loss of this protein is insufficient to induce motor neuron degeneration, which likely involves modifier gain-of-function effects from the hexanucleotide repeat expansion (Lall and Baloh, 2017; Smeyers et al., 2021). Instead, *C9orf72* knockout mouse models primarily result in a systemic autoimmune phenotype, underscoring a shared pathological framework across the major genetic variants of ALS/FTD, in which non-cell-autonomous glial immune dysregulation plays an indispensable role by intersecting with neuronal vulnerabilities (O’Rourke et al., 2016).

## Discussion

### Harnessing TBK1-adaptor complexes for therapeutic selectivity?

The kinase TBK1 represents a particularly challenging therapeutic target given its diverse roles in selective autophagy, endo-lysosomal trafficking, and innate immune signaling in a highly cell-type-specific manner. For the treatment of autoimmune disorders, TBK1 inhibition is being explored as a strategy to suppress excessive activation of interferon signaling (Bodewes et al., 2018; Louis et al., 2018). On the other hand, a central challenge is how to restore specific downstream functions in the setting of TBK1 haploinsufficiency in ALS/FTD. Either introducing exogenous TBK1 through gene therapy delivery methods, such as adeno-associated viral vectors (AAVs), or enhancing the activity of residual TBK1, may help to promote autophagic clearance and endo-lysosomal homeostasis in selectively vulnerable neurons. However, systemically increasing TBK1 activity could inadvertently augment innate immune signaling, such as the cGAS-STING pathway, in myeloid cells (Helgason et al., 2013; Han et al., 2025). Therefore, targeting TBK1 function may require consideration of both cell type and biological context to maximize therapeutic benefit and minimize unintended consequences. For instance, applying CNS-trophic AAV serotypes for targeted delivery with cell-type-specific enhancers or promoters could act to favor expression in vulnerable neuronal populations (Hunker et al., 2025).

TBK1 adaptor proteins (AZI2/NAP1, TBKBP1/SINTBAD, and TANK) determine the recruitment, localization, substrate selection, and pathway specificity of TBK1 kinase complexes, thereby separating them into discrete signaling elements. Moreover, biochemical studies indicate that these proteins vie with each other and with OPTN for binding to the C-terminal CCD2 region of TBK1 (Ryzhakov and Randow, 2007; Goncalves et al., 2011). For instance, a recent elegant study demonstrated that AZI2/NAP1 and TBKBP1/SINTBAD compete with OPTN binding to TBK1 to govern OPTN-dependent selective autophagy (Adriaenssens et al., 2024). Hence, taken together, this framework provides a conceptual rationale for a potential therapeutic strategy in which TBK1 activity could be selectively modulated by targeting individual adaptor proteins to control the relative activation of downstream TBK1-dependent functions, thereby promoting neuroprotective effects. Given that loss of OPTN function contributes to ALS/FTD and that certain TBK1 variants disrupt the binding between TBK1 and OPTN, the OPTN-dependent roles of TBK1 may be especially important for maintaining neuron health with aging. For instance, in the setting of TBK1 haploinsufficiency, one potential strategy would be to modulate AZI2/NAP1, which is implicated in inflammatory processes (Ujevic et al., 2024), to increase the available pool of TBK1 dimers to engage OPTN and restore proteostasis pathways, while preventing maladaptive activation of inflammatory responses. In general, enhancing TBK1 involvement in selective autophagy and bolstering interactions with autophagy cargo receptors may increase clearance of protein aggregates in neurodegeneration. In contrast, selectively inhibiting TBK1 kinase complexes that engage TBK1 in innate immune signaling could represent a strategy to reduce chronic inflammation. Although this concept has not yet been examined in ALS/FTD models, recent studies in other biological contexts collectively support the feasibility of selectively targeting adaptor proteins (Zhu et al., 2018; Vargas et al., 2019; Ujevic et al., 2024; Wu et al., 2026). Future studies to molecularly dissect the role of TBK1 adaptor proteins (TANK, TBKBP1, and AZI2) in endo-lysosomal pathways in the cellular context of neurons, glia, and immune cells will be necessary to guide pathway-selective therapeutic efforts for both autoimmune diseases and neurodegeneration.

TBK1 variants, therefore, illustrate how deficiency of a key protein kinase can lead to disruptions in autophagy and neuroinflammatory pathways in ALS-FTD. Understanding both its conserved roles, such as the regulation of endosomal RAB7, as well as its cell-type-specific roles across the CNS will likely provide insights into mechanisms driving neurodegeneration and pinpoint novel therapeutic targets.

## Supplementary material

SUPPLEMENTARY TABLE S1 Phenotypic Profiling of TBK1 Mouse Models in ALS: tracking proteostasis and microglial activation.
