# Supplementary material for: Minding the gates: dual cell-autonomous and non-cell-autonomous mechanisms of TBK1 in ALS/FTD
Source: Front Neurosci. 2026 Sep 24;20:1941293. doi: 10.3389/fnins.2026.1941293 (PMC13647265; doi:10.3389/fnins.2026.1941293)
Supplement: Supplementary file 1 [file Table_1.pdf]

Publication Year	Article	Mice Models Used	Studied Time Point	ALS Phenotype & Survival	Signs of Endo-lysosomal Dysfunction	Neuroinflammation	Systemic Inflammation	Neurodegeneration
2010	**Mice lacking Tbk1 activity exhibit immune cell infiltrates in multiple tissues and increased susceptibility to LPS-induced lethality** (*J Leukoc Biol.*, Marchlik et al., PMID:20651301)	**Tbk1$^{\Delta/\Delta}$** (Deletion of exon 2, catalytically inactive truncated protein expressed at very low levels)	3 months old	**↓ Survival after LPS challenge**	NA	**↓ IFN-β/RANTES production<br>Impaired IRF3 activation**	**Systemic immune-cell infiltration<br>Monocytosis**	NA
2015	**Regulation of T-cell activation and migration by the kinase TBK1 during neuroinflammation** (*Nat Commun.*, Yu et al., PMID:25606824)	***Tbk1*-TKO** (CD4-Cre-mediated T-cell specific knockout)	4 months old	ND	NA	NA	**Splenomegaly<br>↑ Activated CD4+ and CD8+ T cells**	NA
2018	**TBK1 Suppresses RIPK1-Driven Apoptosis and Inflammation during Development and in Aging** (*Cell*, Xu et al., PMID:30146158)	Model 1: ***Tbk1$^{-/-}$*; *Ripk1$^{D138N/D138N}$*** (RIPK1 kinase-dead knock-in)	Embryonic days (E14.5) up to birth	**RIPK1 kinase-dead mutation rescues embryonic lethality**	NA	NA	**Inflammation prevented by RIPK1 inhibition**	NA
		Model 2: ***Tbk1$^{+/-}$*;*Tak1$^{\Delta M/+}$*** (Myeloid-specific dual heterozygotes)	Up to 24 months old	**Hindlimb weakness**	NA	**RIPK1-dependent microgliosis**	**ND**	**↑ Motor neuron loss<br>↑ Cortical neurons loss**
2019	**Heterozygous Tbk1 loss has opposing effects in early and late stages of ALS in mice** (*J Exp Med.*, Brenner et al.,PMID:30635357)	***SOD1^{G93A}/Tbk1$^{+/-}$***	P50 (early stage) and P120 to end-stage (late stage)	**Earlier onset<br>Slower late progression<br>↑ Late survival**	**P50 : ↑ p62+/GABARAPL1+ puncta vs SOD1^{G93A}<br>P120 : ↓ p62 puncta vs SOD1^{G93A}**	**↓ Microgliosis and astrogliosis (P120)**	NA	ND
2020	**Haploinsufficiency of TANK-binding kinase 1 prepones age-associated neuroinflammatory changes without causing motor neuron degeneration in aged mice** (*Brain Commun.*, Bruno et al., PMID:33005894)	**Tbk1$^{+/-}$** (Heterozygous B6.129P2-Tbk1tm1Aki)	4 and 22 months old	ND	ND	**Premature 'inflamm-ageing' : altered astrocyte and endothelial transcriptomic signature**	ND	ND
2020	**The Loss of TBK1 Kinase Activity in Motor Neurons or in All Cell Types Differentially Impacts ALS Disease Progression in SOD1 Mice** (*Neuron*, Gerbino et al., PMID:32220666)	Model 1: **TBK1^{G217R}** (Heterozygous kinase-dead knock-in)	Up to 24 months old (or end-stage P189 for SOD1 crosses)	ND	NA	NA	NA	ND
		Model 2: **TBK1$^{G217R/G217R}$; TNFR$^{-/-}$** (Homozygous kinase-dead knock-in _Rescued from early lethality)						
		Model 3: **TBK1^{R228H}** (Heterozygous kinase-deficient knock-in)						
		Model 4: **TBK1$^{R228H/R228H}$; SOD1^{G93A}** (or crossed with TBK1$^{G217R/G217R}$)		**Slower progression<br>↑ Survival Earlier onset**	**↑↑ p62 aggregates<br>↑ Ubiquitin puncta<br>↑ Golgi fragmentation**	**↓ Microgliosis and astrogliosis<br>↓ IFN-response genes**	NA	**↑ Accelerated motor neuron loss<br>No TDP-43 pathology**
		Model 5: **TBK1 cKO; SOD1^{G93A}** (TBK1 motor neuron specific deletion)		**Earlier onset<br>Accelerated progression**		**↓ Microgliosis and astrogliosis**	NA	
2024	**A TBK1 variant causes autophagolysosomal and motoneuron pathology without neuroinflammation in mice** (*J Exp Med.*, Brenner et al.,PMID:38517332)	**Tbk1$^{E696K/E696K}$**	6 and 19 months old	**Motor deficits in males (9mo)**	**↑ p62+/GABARAPL1+ inclusions<br>↑ Cathepsin D/LAMP1+ puncta**	ND	ND	**11-13% Motor neuron loss (19mo)<br>No TDP-43 pathology**
2025	**ALS/FTD-linked TBK1 deficiency in microglia induces an aged-like microglial signature and drives social recognition deficits in mice** (*Nat Commun.*, Lenoel et al., PMID:40858618)	Model 1: **Tbk1-MN-KO** (ChAT-Cre × Tbk1$^{fl/fl}$ _ Targets >90% of spinal motorneurons)	3, 4 and 20 months old	ND	**Progressive p62 accumulation in vulnerable motor neurons<br>Stalled autophagosomes<br>↑ GABARAPL1+/Ubiquitin+ puncta in MN (4mo)**	ND	NA	ND
		Model 2: **Tbk1-MN-KO** (VAChT-Cre × Tbk1$^{fl/fl}$ _ Targets 50% of spinal motorneurons)	3, 10, 16, and 20 months old		**p62+ accumulation in 8% of MN (3mo)<br>Stalled autophagosomes<br>↑ GABARAPL1+/Ubiquitin+ puncta in MN (4mo)**			
		Model 3: **Tbk1-μG-HET** & **Tbk1-μG-KO** (Tbk1$^{fl/fl}$; Cx3cr1-Cre _ Deletes Tbk1 selectively in microglia)	4 and 22 months old		ND	**Early shift to a pro-inflammatory aged-like signature**	**Localized SNr CD3^{+}CD8^{+} T-cell infiltration**	
		Model 4: **SOD1^{G93A}/Tbk1-μG-KO**	168 days old	**Typical SOD1 ALS phenotype**		ND vs SOD1 controls	NA	SOD1-driven motor neuron loss

ND: no difference compared with control
NA: not assessed
↑: increased
↓: decreased
